# Radiation Oncology Physics: A Handbook for Teachers and Students

**DOI:** 10.1038/sj.bjc.6604224

**Published:** 2008-03-04

**Authors:** I Rosenberg

**Affiliations:** 1 University College Hospital, London, UK

This book, published in 2005 by the International Atomic Energy Agency, is a comprehensive compendium of all of the topics that should be covered by a radiation oncology physics course, from basic physics to dosimetry, commissioning and quality assurance of equipment, treatment planning and radiation protection and safety. It has an extensive section on brachytherapy, some basic radiation biology and a chapter on special procedures and techniques.

As a handbook, as opposed to a textbook, it contains a large number of useful data tables and basic formulae, but in places it does not go deep enough into the issues that are of current concern in the radiotherapy physics community. It has a comprehensive review of various calibration methods for both photon and electron beams, but it does not reproduce the basic tables of the most recent protocols. It also has a reasonably up to date review of the various radiation distribution algorithms employed by modern treatment planning systems, but it does not discuss the advantages and disadvantages of the various approaches.

In general, it is a little disappointing that such a recent publication is not more focused on new developments in radiotherapy and their implications for the physicists' workload. The section on radiation producing equipment is historically inclusive of somewhat outdated models, but it hardly touches on the newer developments, such as cyclotrons or synchrotrons for proton therapy. Multi-leaf Collimators are not even included in this chapter, despite their wide availability, confining them to the special techniques chapter. Virtual simulators are presented alongside conventional ones, but their impact on the treatment planning process is not discussed. The Intensity Modulated Radiation Therapy Section in the otherwise comprehensive section on special procedures is inadequate. Given its great and wide impact on recent practice, one would have expected the Intensity Modulated Radiation Therapy Section to warrant a chapter of its own, with a detailed discussion on the associated quality assurance and its analysis. References to the up and coming heavy particle therapy, such as neutrons, protons and carbon ions, and their physical characteristics, are very sparse.

In summary, this book is a worthwhile addition to the library of any university or hospital department of medical physics, but has a slightly dated feel despite its age.

